# Classical and nonclassical effects of angiotensin-converting enzyme: How increased ACE enhances myeloid immune function

**DOI:** 10.1016/j.jbc.2024.107388

**Published:** 2024-05-17

**Authors:** Kenneth E. Bernstein, DuoYao Cao, Tomohiro Shibata, Suguru Saito, Ellen A. Bernstein, Erika Nishi, Michifumi Yamashita, Warren G. Tourtellotte, Tuantuan V. Zhao, Zakir Khan

**Affiliations:** 1 Department of Pathology and Laboratory Medicine, Cedars-Sinai Medical Center, Los Angeles, California, USA; 2 Department of Biomedical Sciences, Cedars-Sinai Medical Center, Los Angeles, California, USA; 3 Department of Physiology, São Paulo School of Medicine, Universidade Federal de São Paulo, Sao Paulo, Brazil; 4 Research Oncology, Gilead Sciences, Foster City, California, USA; 5 Institute for Myeloma & Bone Cancer Research, West Hollywood, California, USA

**Keywords:** angiotensin II, angiotensin-converting enzyme, macrophage, neutrophil, cancer, immunology, infectious disease, atherosclerosis, Alzheimer’s disease

## Abstract

As part of the classical renin-angiotensin system, the peptidase angiotensin-converting enzyme (ACE) makes angiotensin II which has myriad effects on systemic cardiovascular function, inflammation, and cellular proliferation. Less well known is that macrophages and neutrophils make ACE in response to immune activation which has marked effects on myeloid cell function independent of angiotensin II. Here, we discuss both classical (angiotensin) and nonclassical functions of ACE and highlight mice called ACE 10/10 in which genetic manipulation increases ACE expression by macrophages and makes these mice much more resistant to models of tumors, infection, atherosclerosis, and Alzheimer’s disease. In another model called NeuACE mice, neutrophils make increased ACE and these mice are much more resistant to infection. In contrast, ACE inhibitors reduce neutrophil killing of bacteria in mice and humans. Increased expression of ACE induces a marked increase in macrophage oxidative metabolism, particularly mitochondrial oxidation of lipids, secondary to increased peroxisome proliferator-activated receptor α expression, and results in increased myeloid cell ATP. ACE present in sperm has a similar metabolic effect, and the lack of ACE activity in these cells reduces both sperm motility and fertilization capacity. These nonclassical effects of ACE are not due to the actions of angiotensin II but to an unknown molecule, probably a peptide, that triggers a profound change in myeloid cell metabolism and function. Purifying and characterizing this peptide could offer a new treatment for several diseases and prove potentially lucrative.

## The rise of angiotensin II

While study of the renin-angiotensin system (RAS) began with Robert Tigerstedt’s 1898 identification of renin in the kidney, it was Harry Goldblatt’s 1934 finding that hypertension was induced by clamping the renal artery which triggered a burst of biochemical research in the field (1, 2). By 1957, investigators had identified the protein angiotensinogen as the substrate for the aspartyl protease renin, and that the product, the ten amino acid peptide angiotensin I, was itself further cleaved to the eight amino acid peptide angiotensin II (Ang II) by the zinc-dependent dicarboxypeptidase angiotensin-converting enzyme (ACE). This is often referred to as the classical RAS. While this review predominantly concerns ACE, we note that Ang II is a remarkable molecule that, after binding to its major cell surface receptor termed angiotensin II type 1 (AT1), has effects throughout the body: brain (increased thirst), gut (increased salt absorption), adrenal gland (aldosterone release), nerves (increased sympathetic activity), vascular smooth muscle (constriction and increased vascular resistance), heart (increased cardiac output), and kidney (increased salt reabsorption) (3). The result is increased blood pressure. The story of ACE, Ang II, and blood pressure even has an appearance by a snake (*Bothrops jararaca*) which produces a peptide in venom that inhibits ACE and helped lead to the development of the first prescribed ACE inhibitor (ACEi) captopril in 1981 (3, 4). ACEis block production of Ang II and are used to treat hypertension, heart failure, and kidney disease. Indeed, the results of the 1987 CONSENSUS heart failure clinical trial are still shocking: a 31% reduction of mortality at 1 year in a group of severe heart failure patients administered an ACEi as compared to those given placebo (5). ACEis, the product of 53 years of biochemical investigation, have extended life for millions. But understanding and treating hypertension, and by extension preventing stroke and cardiovascular disease, was always the goal and Ang II was always the key. Leonard Skeggs, for example, purified Ang II in 1956 using a bioassay in which the activity of countercurrent fractions was assessed for their ability to increase rat blood pressure (6, 7). Ang II has been widely studied; a PubMed search gives more than 77,000 entries.

## How Ang II affects cancer

**Figure 1:**
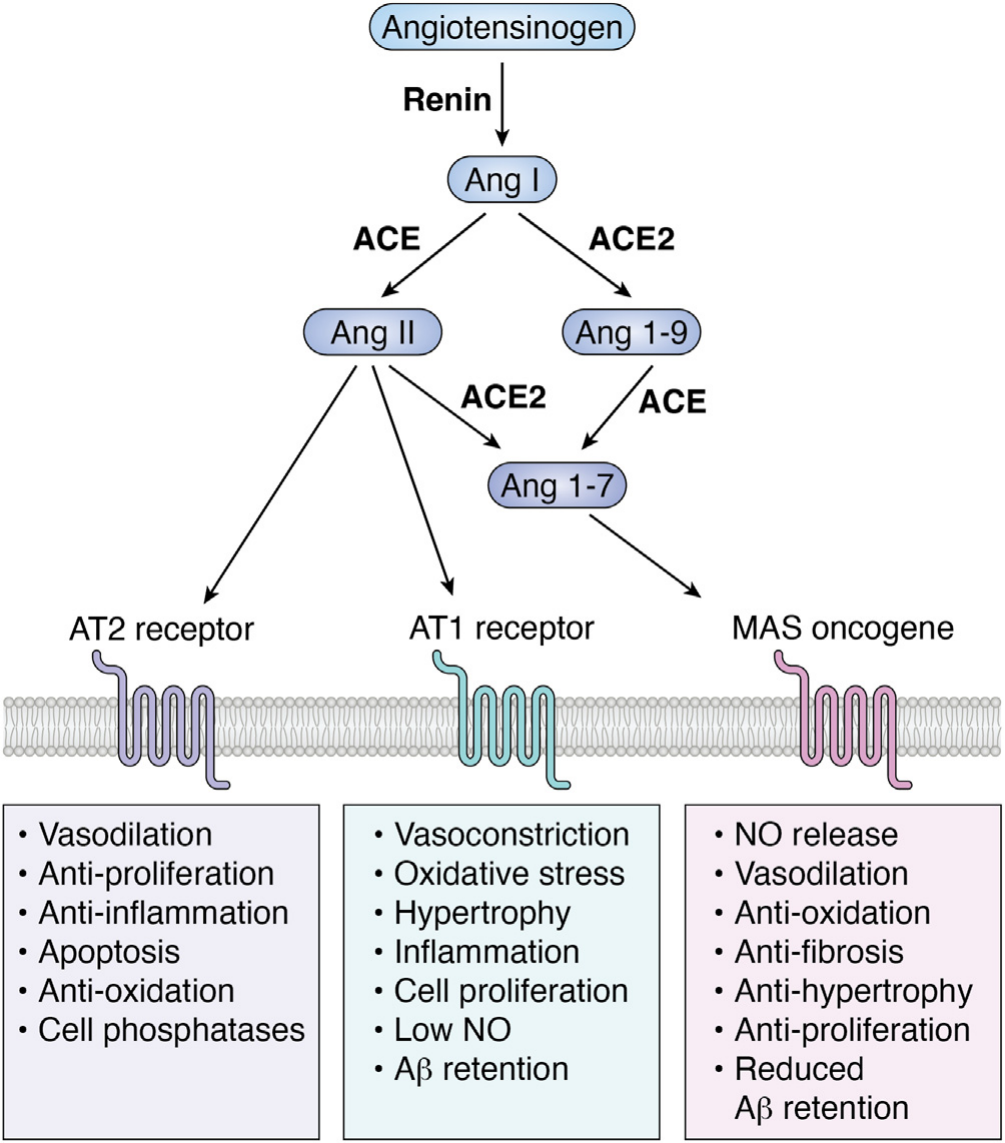
**A simplified version of the renin-angiotensin system.** The protein angiotensinogen is cleaved by renin to release the amino terminal ten amino acids, a peptide called angiotensin I (Ang I). This is further degraded by ACE or ACE2 into Ang II or Ang 1 to 7. Ang II binds to the AT1 receptor–inducing vasoconstriction and many other effects that raise blood pressure (3). It also has a variety of cellular effects including cell proliferation and the induction of inflammation. The AT2 receptor is found in fewer adult tissues than the AT1 receptor; Ang II binding to this receptor counteracts the effects of the AT1 receptor. Ang 1 to 7 is a vasodilator that, by binding to the Mas oncogene, also counteracts the effects of the AT1 receptor (9, 14, 38, 130). AT1, angiotensin II type 1; ACE, angiotensin-converting enzyme; Ang, angiotensin.

While the original formulation of the RAS included two enzymes and two peptides, it is now clear that the totality of the RAS is far more complex, involving many more peptides, enzymes, and cell surface receptors than originally envisioned (8, 9). Conceptually, the biggest advance was the discovery of the ACE homolog ACE2 (10, 11). While ACE2 is now recognized as the receptor for the severe-acute-respiratory-syndrome-related coronavirus and severe-acute-respiratory-syndrome-related coronavirus 2 coronaviruses, the finding that the carboxypeptidase ACE2 converts Ang II to the vasodilatory peptide angiotensin 1 to 7 (Ang 1–7) led to envisioning the RAS as a reciprocal two-axis system in which ACE/Ang II/AT1 receptor causes increased blood pressure, inflammation, and fibrosis while being opposed by the ACE2/Ang 1 to 7/Mas oncogene axis which typically has the opposite effects (Fig. 1) (12, 13, 14, 15, 16). Also, the binding of Ang II to a receptor termed AT2 typically opposes the actions of AT1 receptor binding (17). Given enhanced cell growth, invasion, angiogenesis, and inhibition of apoptosis by the Ang II/AT1 axis, there is an extensive literature on the RAS and cancer (9, 18, 19). This subject can be divided into three areas: possible promotion of tumors by Ang II, effects of ACEis in noncancer patients, and blocking the RAS as a means of preventing or ameliorating cardiotoxicity associated with anticancer treatment.

Several reviews have recently discussed Ang II and the promotion of cancer (9, 20, 21, 22, 23, 24, 25). The Ang II/AT1 axis promotes activation of a number of proteins and pathways associated with cell proliferation including epidermal growth factor receptor activation, Janus kinase-signal transducers and activators of transcription signaling, and mitogen-activated protein kinase activation (26, 27, 28). Ultimately, it may be the activation of some combination of the protein kinase B/mammalian target of rapamycin (mTOR), signal transducers and activators of transcription 3, and extracellular signal-regulated kinase 1/2 pathways that promote cellular proliferation (9, 27, 29). Ang II binding to the AT2 receptor can oppose these activities *via* phosphatase activation (18). Similar opposition to AT1 stimulation is seen after Ang 1 to 7 binding to the Mas receptor which can have antiproliferative effects by inhibiting extracellular signal-regulated kinase 1/2 and PI3 kinase/protein kinase B signaling (9). Selective activation of the AT2 receptor has been suggested as therapy for human prostate cancer based on rat studies where the AT2 agonist compound 21 reduced both androgen receptor expression and the proliferative activity of prostate cancer cells in a rat model of disease (30).

Most patients taking ACEis do so for blood pressure control or other cardiovascular problems and not for cancer. Since hypertension is a life-long disease, such patients often take ACEi for decades. In 2018, an analysis of a United Kingdom cohort of nearly 1 million adult hypertensive patients treated with antihypertensive drugs reported that ACEi were associated with increased risk of lung cancer as compared to patients treated with an Ang II AT1 receptor antagonist (ARB) (31). With ARB therapy, ACE remains fully active. The lung cancer association increased with duration of ACEi drug treatment, first becoming significant in patients after 5 years of therapy and increasing up to 10 years of drug administration. These observations have been controversial and have led to additional analyses by meta-analysis (*i.e.*, pooling) of clinical data (32, 33). The most recent study included records from 13 million patients and concluded “ACEI usage is a greater risk factor for lung carcinogenesis than angiotensin receptor blocker use, especially in Asian patients.” (33) No mechanism was suggested by the authors. Below, the effects of ACE on the immune response of mice to tumors are discussed including the finding that myeloid ACE expression enhances antitumor immunity.

Advances in chemotherapy have significantly altered the modern treatment of cancer, but drugs such as the anthracycline doxorubicin, trastuzumab, and Cisplatin can be cardiotoxic (34, 35). Postulated mechanisms include free radical generation and DNA topoisomerase II ß inhibition. Both ACEi and ARBs are being investigated as prophylactic treatments to reduce or prevent such damage (36, 37).

## The role of the RAS in Alzheimer’s disease

Alzheimer’s disease (AD) and other neurodegenerative diseases are a growing problem around the world. While there is universal agreement that brain β-amyloid (Aβ) accumulation is associated with and promotes AD, the precise sequence of events leading to disease is still not understood. What is acknowledged is that hypertension contributes to both vascular dementia and AD (38, 39). In particular, a number of studies have focused on the potential utility of ACEi and ARBs in slowing the onset of dementia. Apart from blood pressure reduction, these agents have several other effects. In rats, ARBs reduce neuroinflammation and appear to induce enzymes such as insulin-degrading enzyme and neprilysin that degrade Aβ (40). By blocking the Ang II/AT1 receptor axis, ARBs promote Ang II binding to the AT2 receptor and the conversion of Ang II to Ang 1 to 7. The net effect is increased nerve cell projections and postulated facilitation of axonal regeneration. There is also reduced oxidative stress, increased nitric oxide, and reduced amyloid deposits (38, 39).

The effects of ACEi are more complex in that these agents have the positive effects of reducing neuro-vasoconstriction and blood pressure, but they also block the catalytic effects of ACE which can degrade neurotoxic Aβ_1–42_ into the less toxic Aβ_1–40_ (41, 42). Further, ACEi reduce the formation of Ang 1 to 7 and the hexapeptide Ang IV which promote neurologic health (38, 39). The dichotomy between the predominantly advantageous effects of ARBs and the mixed effects of ACEi is reflected in several clinical studies comparing the neuroprotective effects of ACEi *versus* ARBs; most such studies have found a distinct advantage of ARBs over ACEi (38, 39). For example, a recent retrospective clinical study of 57,420 patients with heart disease in South Korea found that long term use of ARBs that cross the blood-brain barrier was associated with a significantly reduced risk of AD development as compared to individuals not using such drugs (43). No such association was found for ACEi (including ACEi that cross the blood-brain barrier) and indeed the direct comparison of ARBs to ACEi found that ARBs were superior to ACEi in reducing AD risk.

Below, we discuss studies in mice which show a direct effect of myeloid cell ACE expression with the immune response of such cells. Mice that are genetically prone to develop “Alzheimer’s-like” disease have much less disease when their immune cells make large amounts of ACE.

## A Gedankenexperiment

In any review of the RAS or ACE, there will inevitably be discussion of the many physiologic effects of Ang II. Indeed, Ang II is so central to blood pressure control and cardiovascular function, and hypertension and heart failure are such widespread and serious problems that they dominate how people conceptualize the RAS. Verily, ACE does convert the inactive precursor Ang I to Ang II, and thus when people think of ACE, they think of Ang II. It is the blinding sun.

But what of the moon and stars, by which we mean that ACE has wide substrate specificity and cleaves many other peptides besides Ang I. A few, such as bradykinin, substance P, and acetyl-seryl-aspartyl-lysyl-proline (SDKP), are well studied but recent investigation indicates that ACE can cleave scores of other peptides ranging from tripeptides to Aβ_1–42_ (42, 44). Consider a Gedankenexperiment (thought experiment/hypothetical situation) in which Goldblatt, who served in World War I, does not publish his 1934 paper and that the first association of ACE and disease is the 1975 observation by Jack Lieberman of elevated ACE serum levels in sarcoidosis patients (45). A pulmonologist, Lieberman found that ACE levels mirrored the activity of the disease: increasing with disease activity and returning to basal levels in treated patients. Sarcoidosis is a disease characterized by granuloma, an inflammatory aggregate composed of macrophages, giant cells (multinucleated cells that are formed from macrophages), and a surrounding collar of lymphocytes. It is the macrophages and giant cells of the granuloma that make high levels of ACE. In fact, lesional macrophages and myeloid derived giant cells in virtually all human granulomatous diseases (histoplasmosis, leprosy, nonnecrotic “miliary” tuberculosis, granulomatosis with polyangiitis) express abundant ACE (46, 47, 48, 49). Granuloma is an ancient form of immune response to persistent stimuli (50), and ACE production by macrophages in these diseases has nothing to do with blood pressure.

ACE expression by macrophages extends beyond granuloma. Extensive study of human atherosclerotic blood vessels shows that macrophages identified in both early and late stage disease plaques also make abundant ACE (51, 52). When human peripheral blood monocytes differentiate to macrophages, cell ACE activity increases 9-fold (53). In mice, ACE expression in neutrophils, macrophages, and dendritic cells (an antigen presenting cell) rapidly increases following *Staphylococcus aureus* or *Listeria* infection (54, 55). Thus, the thought experiment might give rise to a world where ACE is considered part of the immune response. Until very recently, there was no hypothesis about why myeloid cells participating in an immune response make increased ACE.

## Elevated macrophage ACE increases the antitumor response

**Figure 2:**
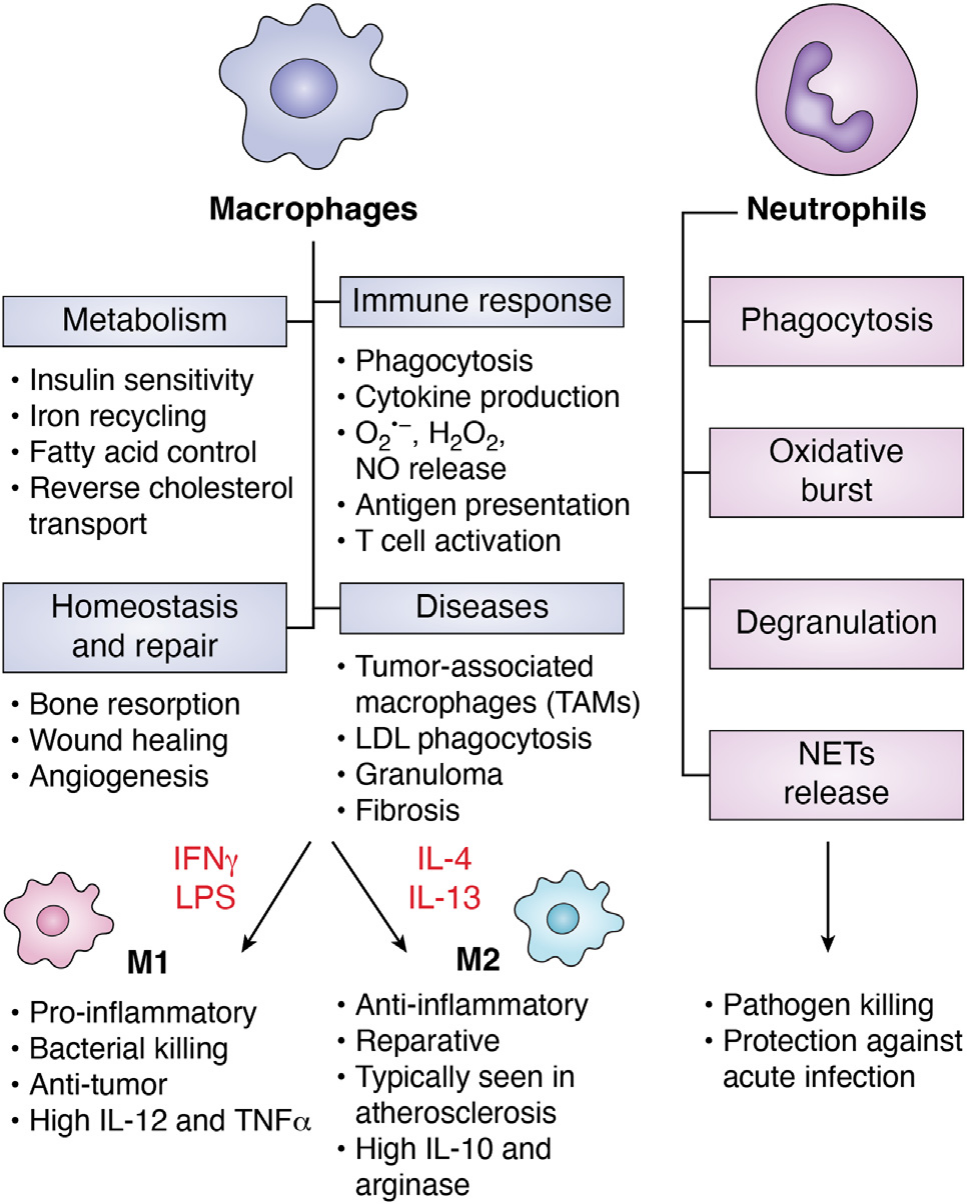
**Different functionality of myeloid cells** (57, 131)**.** Macrophages are phagocytic and anti-bacterial. In addition, they play key roles in physiological mechanisms that affect metabolism, homeostasis, and tissue repair. Macrophages can contribute to several diseases such as cancer and atherosclerosis. Two subsets of macrophages, M1 and M2, indicate different activation states and exert different functions in health and disease. Lipopolysaccharide (LPS) and interferon γ (IFNγ) induce M1 polarization. M1 macrophages secrete proinflammatory cytokines such as interleukin-1 (IL-1), IL-6, IL-12, tumor necrosis factor α (TNFα), and inducible nitric oxide synthase (iNOS) that will kill pathogens and malignant tumor cells. On the other hand, cytokines such as IL-4 and IL-13 induce M2 polarization of macrophages which participate in tissue repair and immunosuppression by secreting IL-10 and arginase (132). M2 macrophages are involved in wound healing and angiogenesis in addition to playing a key role in atherosclerosis. Neutrophils use several mechanisms to kill pathogens including phagocytosis, production of reactive oxygen molecules and hypochlorite, release of antibacterial proteins that are stored in granules, and neutrophil extracellular traps (NETs) composed predominantly of DNA.

Myeloid cells represent a large family of related cell types that include macrophages and neutrophils, cells recognized for their ability to take up and destroy unwanted material such as infectious agents and live or dead cells (Fig. 2) (56). Macrophage function is more complicated than just phagocytosis, and it even goes beyond the concept of immunity as modern research has implicated these cells in many different cell processes such as development, metabolic homeostasis, tissue repair, cancer development, and more (57). The interplay of macrophages and metabolism is complex (58). For example, there are instances in which bone marrow transplantation from genetically modified mice have induced significant changes in the whole body metabolism of recipient mice (59, 60, 61). Perhaps more interesting is that phenotypic variations in macrophage function (*e.g.*, M1 *versus* M2 macrophages) are associated with different internal metabolic states. As example, studies in mice indicate that the activation of macrophages by lipopolysaccharide is associated with a transition of ATP production from oxidative metabolism to glycolysis (62, 63). Further, mitochondria are now known to have abundant effects on macrophage function (64).

The realization that ACE affects the myeloid immune response originated from studies of a mouse model in which targeted homologous recombination in embryonic stem cells was used to place expression of the natural ACE gene under the control of the *c-fms* promoter (c-fms protein encodes the receptor for macrophage colony-stimulating factor) (65, 66). This was the 10th genetically modified mouse made in the laboratory and thus an animal homozygous for this mutation was named ACE 10/10 (67). In mice and humans, there is normally high ACE expression by lung and kidney epithelium, but in the ACE 10/10 mouse, transcription from the *c-fms* promoter leads to virtually no ACE expression by lung or renal epithelium. Rather, there are markedly increased ACE levels in monocytes and macrophages with ACE 10/10 mice expressing from 15- to 25-fold natural levels of ACE depending on how and under what circumstances ACE is assessed. While this mouse has virtually no endothelial ACE expression, the animal’s blood pressure is normal due to the ability of the kidney to maintain homeostasis through regulated renin production (67).

**Figure 3:**
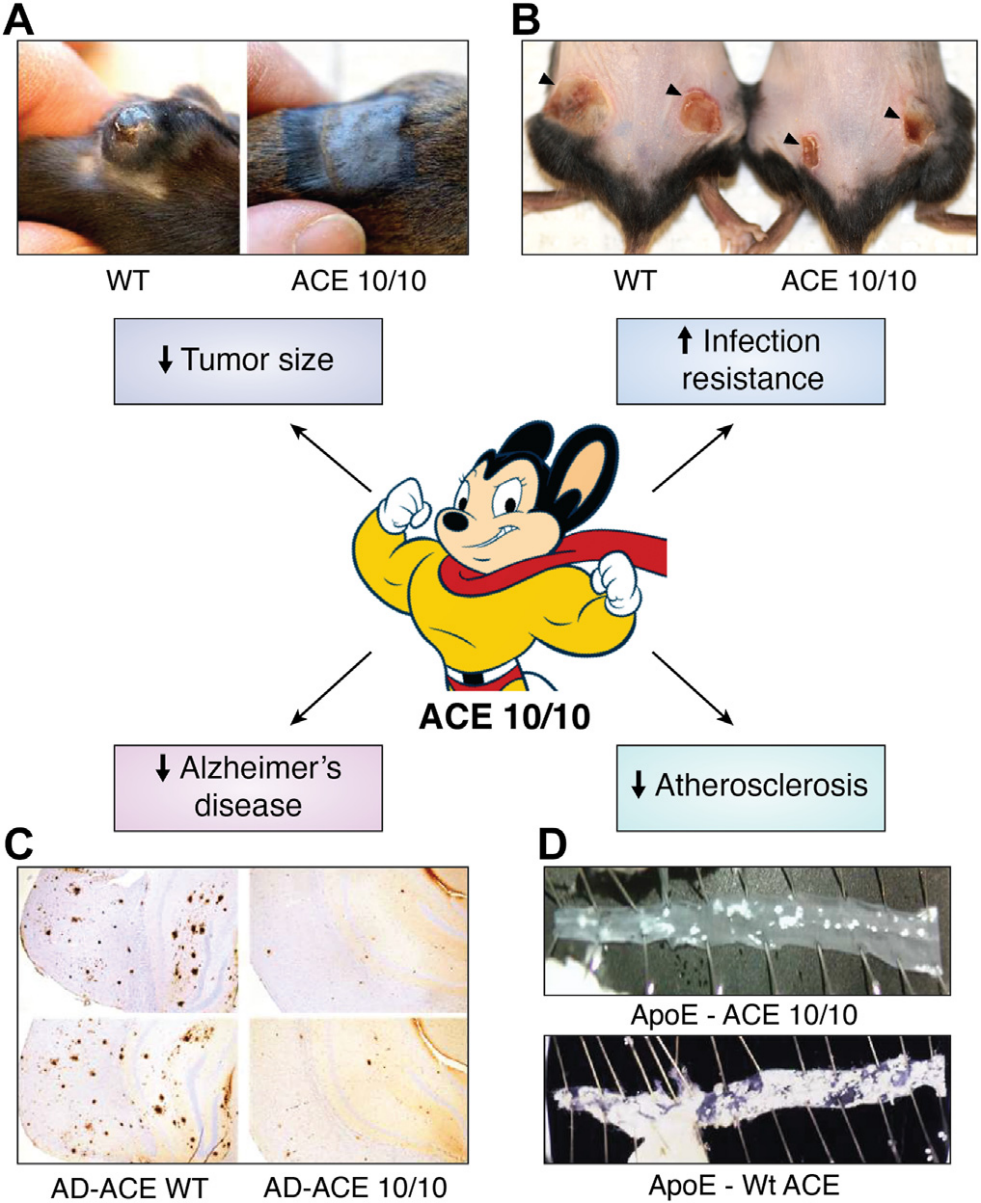
**Changes in the immune response induced by increased ACE expression by macrophages.***A*, B16 melanoma tumor 2 weeks after subcutaneous injection of tumor cells in WT and ACE 10/10 mice. *B*, MRSA lesions 3 days after subcutaneous injection of bacteria in WT and ACE 10/10 mice. *C*, the brains of 8.5 month old AD^+^ACE WT and AD^+^ACE 10/10 mice stained for human Aβ. *D*, the aortas of ApoE KO ACE WT and ApoE KO ACE 10/10 mice 8 weeks after feeding an atherogenic diet and the induction of hypertension. The white-stained material is atherosclerotic disease. Aβ, β-amyloid; ACE, angiotensin-converting enzyme; AD, Alzheimer’s disease; Ang, angiotensin; MRSA, methicillin-resistant *Staphylococcus aureus*.

The response of the ACE 10/10 mouse was studied following several different immunologic challenges. For example, when the mice were challenged with skin implantation of the B16 mouse melanoma tumor line, tumor growth was remarkably suppressed (Fig. 3*A*) with tumor size in ACE 10/10 mice averaging only 17% that in WT mice. Similar results were found with different substrains of B16, different genetic backgrounds of mice bearing the ACE 10/10 mutation, and even WT mice transplanted with ACE 10/10 bone marrow. A metastasis assay where intravenous B16 injection gives rise to lung tumors showed that the number of tumors in ACE 10/10 mice averaged 34% the number in WT mice (68). What makes all these findings particularly striking is that the immune defense against B16 tumor growth is predominantly mediated by T cells, not macrophages (67). This implies that the interaction between macrophages and T cells in ACE 10/10 mice is more effective than in a WT mouse and, in fact, detailed analysis of T cells showed that ACE 10/10 mice develop more antitumor T cells in response to tumor challenge.

How macrophages interact with T cells has been addressed by others (69, 70). An important part of this interaction is intracellular editing of peptides by myeloid cell peptidases associated with the loading of these peptides onto major histocompatibility (MHC) class I or class II proteins followed by surface presentation of the peptide–MHC complex to T cells (71, 72). In macrophages, ACE edits both MHC class I and class II peptides, and the resulting change in displayed peptides may be a part of the reason for the difference between the antitumor response in ACE 10/10 and WT mice (54, 73).

The response to B16 melanoma was studied in three other lines of mice, each transgenic for a construct in which the *c-fms* promoter drives ACE expression in myeloid cells (74). ACE contains two independent catalytic domains that are termed the N and C domains. One line of transgenic mice was made using a construct containing WT ACE (Tg-ACE mice) in which both ACE domains were catalytic. In contrast, the version of ACE in the other two lines contained zinc binding region point mutations selectively eliminating catalytic activity by either the ACE N-domain (Tg-NKO) or the C domain (Tg-CKO). The three lines of mice had roughly equivalently expression of ACE in macrophages and neutrophils. Both the mice expressing increased WT ACE (Tg-ACE) and the Tg-NKO mice having an active C domain showed reduced B16 tumor growth (16% and 23% of WT tumor volume) similar to what is seen in ACE 10/10 mice. However, the Tg-CKO mice, lacking catalytic activity by the ACE C domain, had tumor growth of 85% that observed in control WT mice. Thus, *in vivo*, it is the ACE C domain that is critical for the enhanced antitumor activity of ACE in myeloid cells.

As discussed, the most studied substrate of ACE is Ang I and, *in vivo*, it is the ACE C domain that is predominantly responsible for producing Ang II (75). There are four ways to inhibit Ang II in a mouse: genetically eliminate angiotensinogen, block renin activity with a specific inhibitor, block ACE with a medicinal inhibitor, or block the AT1 receptor (the major Ang II receptor). All four approaches were used and only an ACEi blocked the increased immune response typical of increased myeloid ACE expression (55, 67, 74). Also, infusing Ang II into a mouse does not cause the ACE effect of increased immunity (55). Given that angiotensinogen is the source of Ang I (and thus all subsequent degradation products such as Ang 1–7), these data imply that the response to increased ACE expression is not mediated by any Ang peptides. Finally, neither a bradykinin B2 receptor blocker nor a substance P neurokinin 1 receptor blocker inhibited the ACE effect of increased myeloid function (55). In other words, the peptide product responsible for the effects of ACE on myeloid cells is unknown and perhaps even undiscovered. As we discuss below, such a peptide may have effects on diseases quite removed from an antitumor response.

## Reduced infection in ACE 10/10 mice

Defense against tumors is often associated with macrophages that are aggressively proinflammatory, a phenotype sometimes termed M1 (76). A similar phenotype is often exhibited in response to acute infection. However, while the B16 tumor model is an adaptive immune response dependent upon antigen presenting cells and T cells, many infection models test the rapid, innate response of the immune system. One such model is the response to infection by methicillin-resistant *S. aureus* (MRSA) where a typical assay is to infect mice by an intradermal injection of MRSA and then to measure the daily size of the lesion and the number of bacteria within the lesion at sacrifice a few days after infection (77). When this assay was performed, the skin lesions in ACE 10/10 mice were 22% the size and contained roughly 50-fold fewer viable bacteria than lesions in WT mice (Fig. 3*B*) (78). These results are particularly significant because mice are much more resistant to MRSA than humans. Nonetheless, the immune response of ACE 10/10 mice was markedly better, indicating a more vigorous immune response than that possibly achievable by WT mice. When ACE 10/10 mice were further tested by intravenous injection of the gram-positive organism *Listeria monocytogenes*, they again demonstrated better resistance than WT animals with about 15% fewer splenic bacteria (78).

The biochemistry used by myeloid cells to kill bacteria has been well studied (79, 80). A critical feature is superoxide production by these cells. It is perhaps not surprising that when stimulated with phorbol 12-myristate 13-acetate, macrophages from ACE 10/10 mice produce more superoxide than WT mice (roughly 2.5-fold after 20 min) (55). There is also evidence that ACE 10/10 macrophages produce more inducible nitric oxide synthase (2-fold) and reactive nitrites (2.2- to 2.9-fold) following stimulation (78).

## Reduced AD and atherosclerosis in ACE 10/10 mice

One of the functions of macrophages is to eliminate detritus, such as that following infection or in some chronic diseases. While it is perhaps a simplification to link AD and atherosclerosis, important to the pathology of both diseases is the inability to eliminate unwanted deleterious material: Aβ that comprises AD plaques and lipid from blood vessel walls which fills macrophages leading to “foam cells” that contribute to atherosclerotic plaques (81). For both AD and atherosclerosis, there are mouse models that mimic aspects of the human disease, allowing one to investigate the effect of enhanced macrophage function in illnesses responsible for much human morbidity.

To test a model of AD, mice genetically modified to overexpress the pathogenic peptide Aβ_1–42_ (AD^+^) were used (82). The experiment compared such AD^+^ mice having normal ACE expression to equivalent AD^+^ mice that are also ACE 10/10. After 8.5 months of age, histologic evaluation of brain sections for Aβ plaques showed that the AD^+^ACE 10/10 mice had only 21% to 31% the cortical and hippocampal brain plaque area present in AD^+^ mice with WT myeloid ACE expression (Fig. 3*C*). In this model, disease is associated with learning defects which were evaluated through a standardized learning protocol, the Barnes maze, which evaluates mouse spatial learning and memory (83). Using this, AD^+^ animals were slower to learn and have decreased memory retention than non-AD control mice. In contrast, AD^+^ACE 10/10 mice retained cognitive ability to the point where they were not statistically different from non-AD mice. Others have shown that ACE is able to slowly cleave Aβ_1–42_ (41, 42). Whether increased enzymatic cleavage of this peptide is the explanation for the reduced pathology or whether it is due to a more generalized effect of enhanced myeloid cell function is not known. Further, the brain contains microglias, which are resident myeloid cells akin to macrophages. In the ACE 10/10 animals, microglia make somewhat more ACE than these cells in a WT mouse (82). It is possible that this contributes to reduced disease in the AD^+^ACE 10/10 mice. Analysis of how effective microglia might be in AD if they express increased ACE is still being studied. A major conclusion of the AD studies is that better macrophage function is not only advantageous against obvious immune challenges such as infection but also significantly lessens a disease of abnormal peptide deposition in which the immune response of WT mice is ineffective in clearing Aβ and is actually neurotoxic. Whether this is also true in humans remains an open question, though some have suggested a protective effect of cerebral ACE (84).

As discussed, macrophage dysfunction is thought to play a major role in the pathogenesis of atherosclerosis (85). Specifically, the natural process by which macrophages recycle vascular wall lipids to the liver goes awry with the formation of lipid engorged macrophages termed foam cells. Such cells are unable to egress from the vascular wall and ultimately die releasing cellular contents which promote vascular atherosclerotic plaques. The role of myeloid ACE in two standard mouse models of atherosclerosis was examined. One model is ApoE KO mice fed a high-fat diet. Specifically, ApoE KO mice that were also bred to be ACE 10/10 (*i.e.*, such mice express increased ACE by macrophages) were studied and compared to ApoE KO mice with WT ACE levels (Fig. 2*D*) (86). The second model was the administration of a viral vector to induce increased liver expression of proprotein convertase subtilisin/kexin type 9 (PCSK9) coupled with an atherogenic diet (87). Both models gave similar results: reduced aortic atherosclerotic plaque area in ACE 10/10 mice. For example, in the ApoE KO model, animals that were ACE 10/10 had a 48% reduction of aortic plaque area *versus* ApoE KO mice with WT ACE expression. Reduction of plaque area was 64% when both groups were made hypertensive as well as fed a high-fat diet. Further, ApoE KO animals transplanted with ACE 10/10 bone marrow had a 35% reduction of aortic atherosclerosis as compared to mice transplanted with WT bone marrow (86). In the PCSK9 model, the ACE 10/10 mice had a 44% reduction of aortic atherosclerosis compared to WT mice (87).

In contrast to M1 macrophages which are dominant in tumor and infection models, so-called M2 (reparative) macrophages play a prominent role in atherosclerosis (88, 89). In the PCSK9 model, the aortic inflammation from ACE 10/10 mice contained a 35% higher percentage of M2 macrophages than equivalent WT mice, and these cells expressed somewhat higher levels of M2 markers such as interleukin-10 and arginase (87). Thus, it appears that ACE 10/10 macrophage polarization is more exaggerated irrespective of whether disease triggers a dominant proinflammatory M1 response or, as in atherosclerosis, a reparative M2 process.

## Neutrophils expressing increased ACE

In studying the ACE 10/10 mice, there was an intrinsic risk of basing conclusions on a single line of mice. This was somewhat mitigated by characterizing the transgenic mice termed Tg-ACE (discussed above) that expressed increased myeloid ACE. Such mice were similar to the phenotype of ACE 10/10 (74). Also studied was a third line of mice transgenic for the *c-fms* promoter driving ACE expression, but in these animals, the insertional site of the transgene resulted in neutrophils having a roughly 15-fold increase of ACE, while macrophage ACE expression was very close to WT levels (55). Such mice are called NeuACE. Both neutrophils and macrophages are myeloid cells, but the biology of neutrophils is quite distinct from that of monocytes and macrophages. Thus, NeuACE mice, in addition to being a separate mouse line from ACE 10/10, increased ACE expression by a different cell type (neutrophils) and were studied by different investigators from those characterizing ACE 10/10. Finally, NeuACE mice are transgenic which means that the endogenous ACE gene was unchanged and tissues such as lung and kidney made normal levels of ACE.

The classical role of neutrophils is to protect against acute infection. When NeuACE mice were challenged by infection with either MRSA, *Klebsiella pneumoniae*, or *Pseudomonas aeruginosa*, their resistance was much better than WT animals (55). For example, 3 days after subcutaneous infection with MRSA, the lesional area in NeuACE mice was 22% that observed in WT mice and lesion bacterial count was 23% that of WT. This disparity in resistance was due to neutrophils since specific depletion of these cells with anti-neutrophil antibody eliminated the difference between NeuACE and WT mice. Treatment of NeuACE mice with the ACEi lisinopril increased the lesion size and also eliminated any difference between NeuACE and WT animals. Neutrophils kill bacteria by a variety of means, but central is the generation of superoxide by NADPH oxidase (90, 91). Increased ACE expression in NeuACE neutrophils increased NADPH oxidase; superoxide production was more than 2-fold greater than WT cells after MRSA addition (55). These data are in marked distinction to results from mice null for all ACE (ACE KO mice, KO) where MRSA infection resulted in lesional area and bacterial numbers 4-fold and 3.3-fold greater than the results obtained with WT mice, respectively. ACE KO neutrophils also made less superoxide than WT cells following phorbol 12-myristate 13-acetate stimulation.

A model of endocarditis induced by mechanical injury of the aortic valve and infection of the animals with a low dose of MRSA was also studied (92). For ACE KO, WT and NeuACE mice (ACE low, normal, high), the percentage of mice developing endocarditis was 100% (KO), 40% (WT), and 10% (NeuACE). When NeuACE mice were treated with an ACEi and then tested for endocarditis, 80% of animals developed disease while treatment of NeuACE mice with an angiotensin ARB resulted in 20% endocarditis. Also, treatment of WT mice with an ACEi increased the incidence of disease (90%). These data indicate a consistency in the behavior of mouse neutrophils and macrophages: increased expression of ACE is associated with increased immune function.

## The role of ACE in human neutrophils

An obvious question is whether any of the above observations have applicability to humans, particularly since millions of patients take ACEis without catastrophic results. This question is important since most ACEis are taken to reduce blood pressure or treat heart failure, something also achievable using angiotensin receptor antagonists. Unfortunately, a full discussion comparing ACEi and ARBs is beyond this review. Part of the complexity is that ACEi reduce blood pressure which has many positive benefits. For example, heart failure is associated with lung edema and increased lung infections (93). ACEi can reduce lung edema and subsequent infections, making it difficult to determine the exact effect on myeloid function (94, 95).

A very simple clinical trial examined ACE and human neutrophil function (92). Normal volunteers not on hypertensive medication were asked to donate blood three times: at the start of the study, then after 1 week of a standard daily dose of an ACEi, and finally following a 1 week drug washout period. Each blood sample was tested for *in vitro* killing of MRSA and other pathogenic bacteria. In this study, in which each volunteer acted as their own control, a 5 h test of blood killing of MRSA showed 4.8-fold more bacteria in the samples taken after the ACEi as compared to the baseline (no drug) results. Blood killing returned to near baseline values after the drug washout. Linear regression analysis of all whole-blood killing data showed reduced blood killing caused by the ACEi (*p* < 0.0001), as well as reduced intracellular bacterial killing, superoxide production, and total cell reactive oxygen species production. As an example, analysis of intracellular killing of bacteria showed that after 5 h, neutrophils contain about 4.5% of the live bacteria within the cell at time 0. An equivalent analysis of neutrophils obtained after the ACEi showed 37% viable bacteria at 5 h as compared to time 0. Thus, results with ACEi and human neutrophils are similar to what was observed in mice (92).

## ACE increases oxidative metabolism and cellular ATP

**Figure 4:**
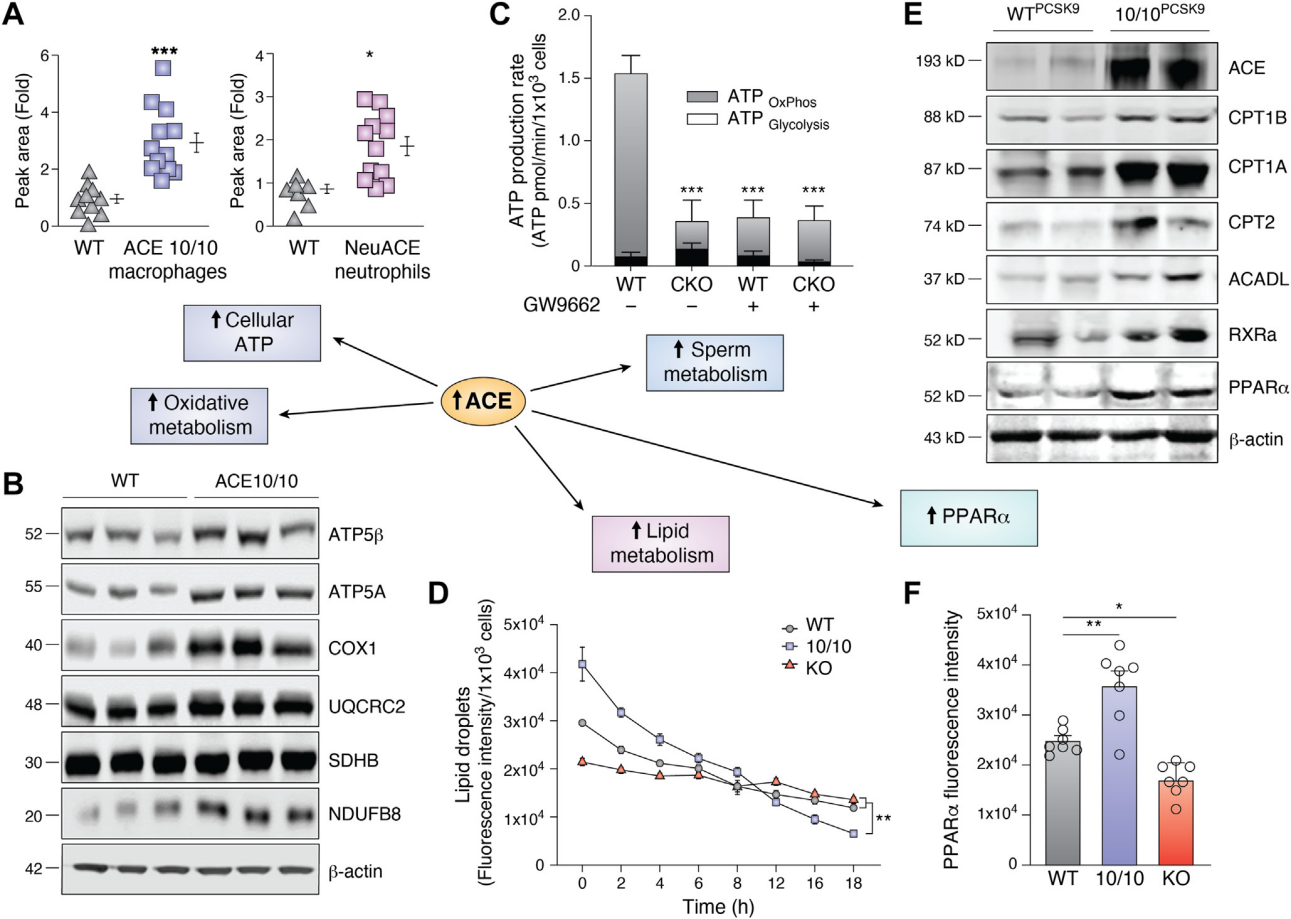
**The cell effects of increased ACE.***A*, the intracellular concentration of ATP was measured by mass spectrometry in ACE 10/10 macrophages and NeuACE neutrophils as well as equivalent WT cells. *B*, Western blot analysis of selected proteins in each of the five electron transport complexes of ACE 10/10 and WT macrophages. ACE 10/10 macrophages have increased amounts of NDUFB8 (complex I), COX1 (MTCO1, complex 4), and both ATP5α and ATP5β (complex 5, ATP synthase) as compared to WT cells. *C*, total ATP production and ATP production due to oxidative metabolism (ATP_OxPhos._) in CKO sperm were about 7-fold less than in WT sperm. Treatment with the PPARγ inhibitor GW9662 eliminated the difference between groups. *D*, WT, ACE 10/10, and ACE KO macrophages were loaded with fluorescent tagged lipid and then, beginning at time 0, levels of intracellular lipid were followed for 18 h. The rate of lipid clearance is reflected by the slope of the lines. ACE 10/10 cells dispose of lipid far more rapidly than the other two groups. *E*, Western blot analysis of aortic macrophages isolated from WT and ACE 10/10 mice following injection of PCSK9-adeno-associated virus and the induction of atherosclerosis. *F*, peritoneal macrophages from WT, ACE 10/10, and ACE KO mice were cultured with oleic acid *in vitro* and then nuclear PPARα was quantified by immunofluorescence with confocal imaging. ACE, angiotensin-converting enzyme; PCSK9, proprotein convertase subtilisin/kexin type 9; PPARα, peroxisome proliferator–activated receptor α.

In both macrophages and neutrophils, the data above establish that increased ACE expression is associated with an increased immune response. To better understand the effects of ACE, mass spectrometry was used to measure the intermediate metabolites of peritoneal ACE 10/10 macrophages, bone marrow derived NeuACE neutrophils and equivalent WT cells (96). ACE 10/10 and NeuACE cells showed a generalized increase in selected cell metabolites. For example, comparing ACE 10/10 and WT macrophages, there were 27 significant differences with 26 metabolites increased in ACE 10/10. NeuACE neutrophils showed a pattern of metabolic changes similar to, but less marked than that seen with ACE 10/10 macrophages. Most surprising, ATP levels per cell were increased in both ACE 10/10 macrophages and NeuACE neutrophils compared to equivalent WT cells (Fig. 4*A*: 3.0-fold and 1.9-fold in macrophages and neutrophils). To verify these data, ATP was chemically measured and showed increases of 2.4 and 1.7-fold in ACE 10/10 and NeuACE cells. This increase was due to ACE catalytic activity as it was reduced by treating mice with an ACEi but not by an ARB. Analysis of ATP levels in macrophages from transgenic Tg-ACE, Tg-NKO, and Tg-CKO mice showed that it is the catalytic activity of the ACE C domain alone that affects cell ATP; macrophages from Tg-ACE and Tg-NKO mice have high ATP levels while cells from Tg-CKO mice have ATP levels equivalent to WT animals (96). Finally, ACE KO macrophages had only about 20% the ATP levels of WT.

Mass spectrometry was also used to study Krebs cycle metabolic intermediates (96). This showed that ACE 10/10 macrophages have significantly higher levels of citrate (3.9-fold), isocitrate (5.2-fold), succinate (1.9-fold), and malate (2.0-fold). Similar results were found in NeuACE neutrophils *versus* WT though the differences were less pronounced, perhaps because neutrophils are more dependent on glycolysis for energy.

Cell respiration of ACE 10/10 and WT macrophages was also measured (96). Under basal conditions, ATP production by glycolysis and oxidative metabolism in ACE 10/10 cells was 31% and 27% higher than in WT cells, respectively. Further, maximal oxygen consumption was 37% higher in ACE 10/10 than in WT macrophages. Additional experiments were performed with macrophages fed glucose, pyruvate/malate, glutamate/malate, or succinate/rotenone. In all instances, oxygen consumption in ACE 10/10 macrophages was higher than WT (for example, 2-fold higher than WT cells with pyruvate/malate). Perhaps not surprisingly, ACE 10/10 mitochondria had a 24% increase in mitochondrial membrane potential while cells from ACE KO mice had a 30% decrease (96). These differences were not due to differences in cell size/number or mitochondrial size/number. Mitochondrial production of ATP is ultimately due to efficient function of the electron transport chain. Western blot analysis with antibodies directed against selected proteins in each of the five electron transport complexes of ACE 10/10 macrophages and NeuACE neutrophils indicated there was a significant increase in the protein from electron transport complex I (NDUFB8) and complex V (ATP5α and ATP5β) as compared to WT cells (Fig. 4*B*). ACE 10/10 macrophages also had an increase of COX1 (MTCO1) which is part of electron transport complex IV (96). These data were confirmed by mass spectrometry.

An increase of electron chain proteins is consistent with the increased respiratory rate found in the ACE 10/10 macrophages, but an important question is whether increased ATP is directly responsible for the increased function of macrophages and neutrophils with increased ACE expression. This is a difficult question to answer with certainty but is important in the sense that metabolic manipulation of ATP levels might suggest a new means of immune enhancement. To examine this, the ATP synthase (complex V) inhibitor oligomycin was used to partially block oxidative phosphorylation (96). This reduced ATP levels in ACE 10/10 macrophages and NeuACE neutrophils to levels equivalent to WT cells, and it also reduced the increased expression of superoxide to WT levels. Not surprisingly, reduced ATP also reduced phagocytosis.

## ACE affects sperm metabolism

The effect of ACE on metabolism may not be limited to just myeloid cells. Some of the highest levels of ACE in the body are present in testicular tissue due to high levels of ACE made by developing sperm (97). Sperm produce a unique, shorter form of ACE called testis ACE that is composed of only one catalytic domain identical to the C domain of the ACE protein made by somatic tissues (termed somatic ACE) (98, 99, 100). Analysis of a variety of ACE genetic models showed that, at least in mice, the lack of ACE, and in particular the lack of ACE C-domain activity, is associated with a marked reduction in male fertility (3). In one study, the mating of WT male mice with WT females produced 19 females with vaginal plugs (indicating copulation), 15 litters, and 153 embryos. In contrast, the mating of ACE C-KO male mice (which lack testis ACE activity) with WT females resulted in 22 females with vaginal plugs, but only 1 litter with one embryo (101). Mechanistically, sperm from mice lacking ACE C-domain activity have several functional defects but perhaps most important is that mature sperm from such animals are not as capable in ascending the female reproductive tract as WT sperm (102). For many years, these data were inexplicable but after the discovery that myeloid cell ACE affects metabolism, it is perhaps not surprising that analysis showed that sperm from animals devoid of ACE C-domain activity have significantly less cellular ATP than WT sperm (32% of WT levels as determined chemically and 11% by mass spectrometry) (103). This is associated with reduced oxidative metabolism (Fig. 4*C*), mobility, and ability for fertilization *in vitro* and *in vivo*. Thus, in both myeloid cells and in developing sperm, catalytic activity of the C domain of ACE is critical for ATP generation in these highly mobile cells. Further, *in vitro* treatment of human and mouse sperm with an ACEi reduced ATP and sperm motility in both cell types, again indicating concordance between ACE function in mice and men (103). Finally, male angiotensinogen KO mice appear fertile, indicating that the effect of ACE on male fertility is due to some other peptide besides Ang II (102). It seems likely that this peptide is similar or identical to the peptide affecting myeloid cell function.

## ACE increases lipid metabolism *via* PPARα

As discussed, histologic analysis of macrophages within human atherosclerotic plaque found high expression of ACE. This phenotype was recapitulated in the PCSK9 mouse model where aortic macrophages from both ACE 10/10 and WT mice expressed more ACEs in the presence of atherosclerotic disease (87). In fact, ACE expression appears to result in a major change in the ability of macrophages to handle lipid. For example, when peritoneal macrophages from ACE 10/10, WT, and ACE KO mice were examined for their ability to take up lipid, the ACE 10/10 macrophages engulfed 140% the amount taken up by WT cells while ACE KO mice showed lipid uptake of only 64% of WT levels (87). To study lipid utilization by macrophages, these cells were loaded with lipid *in vitro* and then observed for an additional 18 h with periodic assessment of intracellular lipid (Fig. 4*D*). The reduction of lipid droplets in ACE 10/10 macrophages was much greater than observed in either WT or ACE KO cells; at 18 h, the ACE 10/10 macrophages contained only 16% of the starting lipid as compared to 41% and 63% for WT and ACE KO cells.

To directly study lipid utilization, peritoneal macrophages from atherosclerotic mice were cultured with either uniformly labeled ^13^C-glucose or ^13^C-oleic acid and 48 h later were analyzed for the percent of cell citrate incorporating the ^13^C label (87). Carbon from both glucose and long-chain fatty acids becomes incorporated into citrate within the mitochondria and thus this assay assesses both the uptake of the labeled reagent as well as its metabolism. There was a striking difference in cells from ACE 10/10 mice compared to those from WT mice, particularly in the handling of lipid. When fed glucose, ACE 10/10 macrophage ^13^C citrate levels were 2.1-fold those of WT cells. However, when fed oleic acid, the levels of ^13^C citrate in ACE 10/10 macrophages were 8.5-fold those of WT cells. The conclusion of these and other experiments is that there is a profound difference in the ability of cells expressing increased ACE to take up and metabolize long-chain fatty acids. Lipids are an abundant source of ATP and thus these data are consistent with the metabolic studies that found high levels of oxidative metabolism and ATP in ACE 10/10 cells.

Given the central role of the transcription factor peroxisome proliferator–activated receptor α (PPARα) in the cellular handling of lipid, the levels of PPARα in aortic macrophages from mice with atherosclerotic disease was evaluated (Fig. 4*E*) (87, 104). This showed approximately 1.9-fold greater expression of PPARα in aortic macrophages than equivalent cells from WT mice. As a transcription factor, PPARα induces dozens of downstream molecules related to lipid metabolism including carnitine palmitoyltransferase (CPT) isoforms 1A, 1B, and 2, which catalyze entry of long-chain fatty acids into the mitochondria for β oxidation (105). Perhaps not surprising, macrophages from ACE 10/10 mice expressed more of these proteins than WT cells (87). Retinoid X receptor α, a binding partner of PPARα, was also increased, as measured by Western blot analysis, but the levels of PPARγ and PPARδ showed no differences between the two groups.

To investigate further the role of ACE and PPARα expression, peritoneal macrophages from WT, ACE 10/10, and ACE KO mice were cultured *in vitro* for 48 h with oleic acid to measure lipid uptake. The mice used in this experiment were not atherogenic in the sense that the mice had not been treated with PCSK9 or a high-fat diet. Here, the use of oleic acid *in vitro* simulates elevated lipid concentrations *in vivo*. Confocal microscopy was used to measure nuclear PPARα. This study found that ACE 10/10 cells expressed 44% more nuclear PPARα than WT cells, while ACE KO cells expressed 31% less than WT (Fig. 4*F*) (87). Western blot analysis of these samples found a similar pattern for expression of CPT1A/B, CPT2, adipose triglyceride lipase, and retinoid X receptor α. In addition, ATP levels in ACE 10/10 macrophages were 1.8-fold and 3.5-fold higher than those of WT and ACE KO cells, respectively. Treatment of ACE 10/10 cells with either the CPT1A inhibitor etomoxir or the PPARα inhibitor GW6471 reduced ATP levels to those of equivalently treated WT cells.

## Other approaches to increase the immune response

Macrophages from ACE 10/10 mice recapitulate (and exaggerate) Leiberman’s 1975 observation of increased myeloid cell ACE within human granuloma, and they may give insight into why increased ACE expression by myeloid cells occurs naturally: increased ACE stimulates oxidative metabolism and cell function. Given the variegated role of myeloid cells (interaction with lymphocytes, antibacterial action, recycling of cholesterol and other lipids, elimination of cellular, and protein debris), this change in function has effects throughout the immune system.

Other groups have investigated ways of increasing myeloid function. For example, diet has been suggested as a means of increasing myeloid function (106). More developed as a locus for drug development are receptors and cell signaling pathways known to regulate myeloid cell function. One such receptor family is toll-like receptors (TLRs) that are key components of the innate immune system (107). The use of natural or pharmacologic stimulators of TLRs has the potential to increase myeloid cell recognition and response to pathogens and to affect the balance of proinflammatory and antiinflammatory cell signals. Several TLR agonists are currently being evaluated. The first FDA-approved stimulating agent was the bacterial strain *Bacillus* Calmette-Guérin (typically used as a vaccine against tuberculosis) which is thought able to stimulate both TLR2 and TLR4 (among several other effects) and has shown efficacy in treating bladder cancer (108, 109, 110). In a murine model, the TLR4 ligand monophosphoryl lipid A (a detoxified form of the lipopolysaccharide), in conjunction with interferon γ, was reported to reprogram tumor-associated macrophages to become tumoricidal (111). There are also attempts to target TLR3, TLR7, TLR8, and TLR9 which are nucleic acid sensors localized in endosomal compartments. For example, the TLR7 agonist imiquimod was approved for the treatment of squamous and basal cell carcinoma (112). Other TLR stimulators include resiquimod (R-848, a TLR7/TLR8 agonist) and motolimod (VTX-2337, a TLR8 agonist) which exhibit promising immunostimulatory activity in different preclinical models (113, 114). In conclusion, TLR targets show potential but at present are not widely used; this area needs further investigation and clinical validation.

The targeting of macrophage “checkpoint” costimulatory and coinhibitory factors are also of great interest. Using agonists or modulators to strategically stimulate the macrophage costimulatory proteins, CD40, CD80, or CD86, can result in heightened phagocytosis, antigen presentation, and proinflammatory cytokine production (115). A well-established target is CD40 and several CD40 agonists are currently undergoing clinical trials as monotherapies or combination therapies. APX005M (sotigalimab), an anti-CD40 agonist antibody, was approved by the FDA as an orphan drug for the treatment of three types of gastrointestinal cancer (116). Conversely, macrophages can suppress T cell activity through coinhibitory factors such as programmed death-ligand 1 and the poliovirus receptor (CD155). Those molecules can be targeted on myeloid cells reversing the T cell suppression (117, 118).

Cytokines also play a role in macrophage activation or inhibition. One example is the enhancement of phagocytosis, an essential defense mechanism against pathogens. Cytokines such as interferon γ and granulocyte-macrophage colony-stimulating factor increase the phagocytic capabilities of macrophages (119). These cytokines also contribute to the enhancement of macrophage antigen presentation which further activates the adaptive immune response.

As we discussed, ACE appears to modify the metabolism of macrophages. Not surprisingly, others have recognized metabolic regulation as loci for macrophage activation. Approaches that convert macrophages from glycolytic to oxidative metabolism have used pathways such as AMP-activated protein kinase and mTOR to increase antimicrobial responses (120). AMP-activated protein kinase activation promotes mitochondrial biogenesis and oxidative phosphorylation, resulting in macrophages with increased energy stores and sustained functionality (121). Similarly, mTOR inhibition shifts macrophages toward a more oxidative state, promoting increased phagocytosis and antigen presentation. Furthermore, lipid metabolism, particularly the balance of fatty acid oxidation and fatty acid synthesis, influences macrophage polarization; activation of PPARs stimulates fatty acid oxidation inducing an M2-like phenotype (122). On the other hand, inhibiting lipid synthesis through acetyl-CoA carboxylase inhibition gives rise to a proinflammatory M1-like state. Thus, in addition to the effects of ACE, the focused regulation of lipid metabolism allows for the improvement of macrophage responses which offers a potential avenue for therapeutic intervention. Together, the strategic regulation of metabolic pathways is potentially a powerful tool in regulating macrophage function.

Finally, we note that epigenetic modifications, such as DNA methylation and histone acetylation, exert profound influence over macrophage phenotypes (123). Targeting these epigenetic modifications was recognized as a novel strategy to enhance macrophage function. For instance, inhibiting DNA demethylation with DNA methyltransferases results in proinflammatory M1-like macrophages (124). Simultaneously, histone deacetylase inhibitors also induce a proinflammatory transcriptional program by maintaining an open chromatin structure (125).

In conclusion, several different approaches are being investigated as a means of regulating macrophage activity. Thus said, the profound metabolic and functional effects of increased ACE expression appear quite unique.

## Future opportunities and challenges in understanding how ACE affects the immune response

In this review, we wish to emphasize two points. First is the remarkable diversity of functions performed by macrophages and other myeloid cells. This becomes apparent from observing the many disease models in which increased myeloid function in ACE 10/10 mice results in reduced disease. Scientists have studied immunosuppression far more than immune enhancement, and what is known about increasing immunity is typically focused on increasing T cell function. What the ACE 10/10 mouse shows is the central role of myeloid cells in the immune response to a wide variety of immune challenges. Importantly, this includes the regulation of T cell function.

Perhaps the most important observation to come from the studies discussed in this review is that the effect of ACE on myeloid cells is not due to the actions of Ang II but due to ACE catalytic activity that either destroys a substrate or, more likely, produces a peptide product that triggers the changes in myeloid biology described here. Indeed, a major reason for writing this review is to bring to wider attention that there is an unknown peptide that has profound effects on myeloid function. While we do not know whether the effects of this on human myeloid cells would exactly phenocopy those seen in the mouse, preliminary data to date suggest that such a peptide may very well have the effect of enhancing human myeloid cell functionality. It appears that *in vivo*, it is the action of the ACE C domain that is responsible for peptide production. Whether this works through a receptor or through some other functional pathway is not known, but peptide isolation would undoubtedly give insight into this. Perhaps more importantly, we wish to emphasize both the commercial opportunity and the medicinal potential of finding the peptide and exploring its mechanism. Myeloid cells play a role in many different diseases and enhancement of myeloid function could very well result in novel approaches to the treatment of several diseases. Isolating a particular peptide is not a trivial task and is not an area of specialization for the group of people who have characterized the ACE 10/10 mouse. It may be that the task of identifying this peptide falls to others. We wish to emphasize to both junior and senior colleagues that identification and commercial development of the intellectual property obtained through such peptide isolation could be highly lucrative. If indeed a single peptide product of ACE is responsible for enhancing the immune response to tumors, infection, and chronic diseases such as atherosclerosis and AD, then having the sequence of the responsible peptide would play into a strength of the pharmaceutical industry, namely, developing a nonpeptide analog. Thus, studies of the ACE 10/10 mouse argue for both the clinical utility of a peptide that markedly enhances myeloid function as well as the commercial bonanza that could result from its purification and identification of its mode of action. The intellectual satisfaction of discovering something so new, potentially powerful, and unexpected speaks for itself.

In terms of other opportunities for the future study of ACE, we note again that Ang II has been extensively studied due to its critical role in blood pressure and cardiovascular function. Important areas for future study are the many other peptides affected by ACE and the many other functions of ACE apart from Ang II production and cardiovascular disease. For example, Trieu *et al.* have studied the role of ACE cleavage of the enkephalin Met-enkephalin-Arg-Phe in the context of addiction (126). Magalhães *et al.* have analyzed the ACE substrate acetyl-SDKP and hematopoietic proliferation, while Ramasamy *et al.* have studied acetyl-SDKP and fibrosis (127, 128). While the wide substrate specificity of ACE was first underlined in 1987, modern tools of peptide analysis should be able to markedly expand the known substrates, products, and roles of ACE (129).

## Conflict of interest

The authors declare that they have no conflicts of interest with the contents of this article.
